# Dapagliflozin improves myocardial flow reserve in patients with type 2 diabetes: the DAPAHEART Trial: a preliminary report

**DOI:** 10.1186/s12933-022-01607-4

**Published:** 2022-09-03

**Authors:** Lucia Leccisotti, Francesca Cinti, Gian Pio Sorice, Domenico D’Amario, Margherita Lorusso, Maria Angela Guzzardi, Teresa Mezza, Shawn Gugliandolo, Camilla Cocchi, Umberto Capece, Luca Indovina, Pietro Manuel Ferraro, Patricia Iozzo, Filippo Crea, Alessandro Giordano, Andrea Giaccari

**Affiliations:** 1 UOC Di Medicina Nucleare, Dipartimento Di Diagnostica Per Immagini, Radioterapia Oncologica ed Ematologia, Fondazione Policlinico Universitario A. Gemelli IRCCS and Università Cattolica del Sacro Cuore, Rome, Italia; 2 Centro Malattie Endocrine E Metaboliche, Dipartimento Di Scienze Mediche E Chirurgiche, Fondazione Policlinico Universitario A. Gemelli IRCCS and Università Cattolica del Sacro Cuore, Rome, Italia; 3 Sezione Di Medicina Interna, Endocrinologia, Andrologia e Malattie Metaboliche, Dipartimento Dell’Emergenza E Dei Trapianti Di Organi (D.E.T.O.), Università Degli Studi Di Bari “Aldo Moro”, Bari, Italia; 4 UOC Di Cardiologia, Dipartimento Di Scienze Cardiovascolari, Fondazione Policlinico Universitario A. Gemelli IRCCS, and Università Cattolica del Sacro Cuore, Rome, Italia; 5 Istituto Di Fisiologia Clinica, Consiglio Nazionale Delle Ricerche (CNR), Pisa, Italia; 6 UOSD Fisica Medica E Radioprotezione, Dipartimento Di Diagnostica Per Immagini, Radioterapia Oncologica ed Ematologia, Fondazione Policlinico Universitario A. Gemelli IRCCS, Rome, Italia; 7 U.O.S. Terapia Conservativa Della Malattia Renale Cronica, Fondazione Policlinico Universitario A. Gemelli IRCCS, Università Cattolica del Sacro Cuore, Rome, Italy

**Keywords:** Diabetes, Metabolism, Myocardial blood flow, Perfusion, PET, SGLT-2

## Abstract

**Objective:**

Cardiovascular (CV) outcome trials have shown that in patients with type 2 diabetes (T2D), treatment with sodium-glucose cotransporter-2 inhibitors (SGLT-2i) reduces CV mortality and hospital admission rates for heart failure (HF). However, the mechanisms behind these benefits are not fully understood. This study was performed to investigate the effects of the SGLT-2i dapagliflozin on myocardial perfusion and glucose metabolism in patients with T2D and stable coronary artery disease (coronary stenosis ≥ 30% and < 80%), with or without previous percutaneous coronary intervention (> 6 months) but no HF.

**Methods:**

This was a single-center, prospective, randomized, double-blind, controlled clinical trial including 16 patients with T2D randomized to SGLT-2i dapagliflozin (10 mg daily) or placebo. The primary outcome was to detect changes in myocardial glucose uptake (MGU) from baseline to 4 weeks after treatment initiation by [(18)F]2-deoxy-2-fluoro-D-glucose (FDG) PET/CT during hyperinsulinemic euglycemic clamp. The main secondary outcome was to assess whether the hypothetical changes in MGU were associated with changes in myocardial blood flow (MBF) and myocardial flow reserve (MFR) measured by ^13^N-ammonia PET/CT. The study was registered at eudract.ema.europa.eu (EudraCT No. 2016-003614-27) and ClinicalTrials.gov (NCT 03313752).

**Results:**

16 patients were randomized to dapagliflozin (n = 8) or placebo (n = 8). The groups were well-matched for baseline characteristics (age, diabetes duration, HbA1c, renal and heart function). There was no significant change in MGU during euglycemic hyperinsulinemic clamp in the dapagliflozin group (2.22 ± 0.59 vs 1.92 ± 0.42 μmol/100 g/min, p = 0.41) compared with the placebo group (2.00 ± 0.55 vs 1.60 ± 0.45 μmol/100 g/min, p = 0.5). Dapagliflozin significantly improved MFR (2.56 ± 0.26 vs 3.59 ± 0.35 p = 0.006 compared with the placebo group 2.34 ± 0.21 vs 2.38 ± 0.24 p = 0.81; p_int_ = 0.001) associated with a reduction in resting MBF corrected for cardiac workload (p = 0.005; p_int_ = 0.045). A trend toward an increase in stress MBF was also detected (p = 0.054).

**Conclusions:**

SGLT-2 inhibition increases MFR in T2D patients. We provide new insight into SGLT-2i CV benefits, as our data show that patients on SGLT-2i are more resistant to the detrimental effects of obstructive coronary atherosclerosis due to increased MFR, probably caused by an improvement in coronary microvascular dysfunction.

*Trial registration* EudraCT No. 2016-003614-27; ClinicalTrials.gov Identifier: NCT03313752

**Supplementary Information:**

The online version contains supplementary material available at 10.1186/s12933-022-01607-4.

## Introduction

Type 2 diabetes (T2D) is associated with an increased risk of cardiovascular (CV) disease [1]. In patients with T2D, large CV outcome trials have shown that sodium-glucose cotransporter-2 inhibitors (SGLT-2i) have beneficial cardioprotective effects [2–7], regardless of the presence of established atherosclerotic CV disease or history of heart failure (HF) [6, 8], with reduction in major adverse CV events and CV deaths, as well as reduced risk of hospitalization for HF, and reduced progression of renal disease and all-cause mortality [9–14]. The multiple mechanisms hypothesized and investigated to explain the beneficial CV effects of SGLT-2i are the subject of continuous and intense debate.

First, SGLT-2i increase fasting levels of ketones and may thus enhance the use of this efficient metabolic fuel in the heart. Furthermore, increased levels of circulating ketones may have potentially beneficial effects on myocardial perfusion, left ventricular ejection fraction (LVEF) and cardiac output [15]. Second, the glycosuric, diuretic and natriuretic effects of SGLT-2i may favorably impact LV function and myocardial contractility by increasing the preload, while reductions in blood pressure and arterial stiffness may reduce the afterload. It has also been suggested that SGLT-2i may differ somewhat from classical diuretics, as they promote a greater reduction in interstitial versus intravascular volume [16]. Finally, the improvement in insulin resistance may be associated, and partly explained, by an increase in myocardial perfusion and/or glucose uptake.

A unifying hypothesis for the complex and interlinked mechanisms responsible for changes in myocardial metabolism and perfusion occurring with SGLT-2i treatment is still missing. Previous data have demonstrated that the SGLT-2i dapagliflozin is able to remove glucotoxicity leading to an improvement in muscle insulin sensitivity [17]. It is well known that T2D is associated with cardiac insulin resistance, thus an amelioration of insulin sensitivity could lead to an increase of myocardial glucose uptake (MGU). Therefore, the aim of this study was to evaluate whether SGLT-2i treatment with dapagliflozin compared to placebo improves glucose metabolism in patients with T2D and coronary artery disease, by measuring MGU and whole-body insulin resistance and to determine whether this improvement is associated with an increase in myocardial flow reserve (MFR). Likely, a better understanding of the mechanisms of action of SGLT-2i in T2D patients could help understand the benefits of SGLT-2i in patients with HF.

For this reason, we conducted a multidisciplinary clinical study on a well-selected population of T2D patients, using Positron Emission Tomography/Computed Tomography (PET/CT) to assess MGU and whole-body glucose uptake (WBGU) during euglycemic hyperinsulinemic clamp, MFR and LVEF. Other secondary outcome analyses, i.e., change from baseline in white adipose tissue and microbiota are still ongoing. For details, please see reference [18].

## Research design and methods

The study design of the DAPAHEART trial has been published recently [18]. The primary outcome was to detect changes in MGU from baseline to 4 weeks after treatment initiation by [(18)F]2-deoxy-2-fluoro-D-glucose (FDG) PET/CT during hyperinsulinemic euglycemic clamp. The main secondary outcome was to assess changes from baseline in myocardial blood flow (MBF) and MFR measured by ^13^N-ammonia PET/CT. The study was approved by the local ethics committee (Fondazione Policlinico Universitario Agostino Gemelli IRCCS, study protocol code GIA-DAP-16-005) and registered at eudract.ema.europa.eu (EudraCT No. 2016-003614-27) and ClinicalTrials.gov (NCT 03313752). Informed, written consent was obtained from all participants.

### Trial design and participants

This was a phase III, single-center, randomized, two-arm, parallel-group, double-blind, placebo-controlled study, consisting of a screening phase (days -14 to -1), a 4-week double-blind, placebo-controlled treatment phase and a 4-week follow-up phase. The placebo tablets were provided by the pharmaceutical company (identical in appearance, taste and smell). The randomization was performed by the hospital pharmacy. Inclusion criteria were (1) T2D, 92) no previous history of myocardial infarction, (3) stable coronary artery disease (coronary stenosis ≥ 30% and < 80% in at least one native major coronary artery), with or without previous percutaneous coronary intervention (> 6 months), with no evidence of critical restenosis and no indication to myocardial revascularization according to the current guidelines of the European Society of Cardiology [19], (4) glycated hemoglobin [HbA1c]: 7–8.5% or 53–69 mmol/mol on stable standard of care anti-hyperglycemic regimen, (5) diabetes duration < 10 years, (6) fasting C-peptide > 1 ng/mL (0.33 nmol/L) at screening visit, (7) age: 40–75 years, (8) body mass index (BMI): 25–35 kg/m^2^, (9) women in surgical or natural menopause or with childbearing potential but unwilling to become pregnant during the study and non-breastfeeding women. Exclusion criteria were (1) type 1 diabetes or previous diagnosis of Latent Autoimmune Diabetes of Adults, (2) use of pioglitazone, loop diuretics or basal-bolus insulin therapy for at least 3 months prior to the screening visit or use of systemic steroids less than 3 days prior to the screening visit, (3) NYHA class III or IV, (3) reduced LVEF (≤ 50%), (4) unstable angina, (5) moderate to severe renal impairment (estimated glomerular filtration rate < 60 mL/min/1.73 m^2^) or overt proteinuria, (6) severe liver dysfunction, (7) contraindications to adenosine administration, (8) acute urinary tract infection, (9) history of breast, bladder or prostate cancer, (10) coronary artery disease with a coronary stenosis ≥ 80% in a major coronary artery defined by invasive coronary angiography, (11) inability to provide informed, written consent. The study design is illustrated in Additional file 1: Fig. S1.

All participants were screened for all applicable inclusion and exclusion criteria, underwent baseline assessments, and were enrolled during the screening phase. Participants were randomly assigned in a 1:1 ratio to receive dapagliflozin (10 mg, orally, once daily) or placebo for 4 weeks. Randomization and packaging of medicine were handled by the hospital pharmacy. Excess trial medication was returned, and the remaining number of capsules was counted to ensure compliance. All baseline assessments were repeated at the end of treatment (4 weeks) and an 8-week follow-up visit was performed to assess safety and clinical parameters.

### PET imaging and analysis

PET/CT was performed at baseline and after 4 weeks of treatment using a 3D PET/CT scanner (Biograph mCT, Siemens Healthcare).

### FDG PET/CT during euglycemic hyperinsulinemic clamp

All PET studies were conducted after an overnight fast. A FDG PET/CT in conjunction with a hyperinsulinemic euglycemic clamp was performed to assess changes from baseline in insulin- stimulated MGU and WBGU. Prior to the PET scan, patients were asked to void their bladder, and a polyethylene cannula was inserted in a superficial forearm vein for infusion of glucose, insulin and FDG. A second cannula was threaded into a superficial vein of the opposite arm or hand, for blood glucose sampling. At time 0, a primed-constant insulin infusion (40 mU·min^−1^·m^2^ of body surface area) was started. The prime consists of four times the final constant rate for the first 4 min, followed by two times the constant rate for 3 min. Euglycemia was maintained using a 20% glucose infusion adjusted according to frequent plasma glucose measurements. Under near-steady metabolic state conditions, the exogenous glucose infusion rate approximates the total amount of glucose metabolized by all tissues, representing an index of whole-body insulin sensitivity or WBGU (expressed in mg/min per kg of body weight). Blood samples for measuring chemistry and hormones (e.g., plasma glucose, insulin, C-peptide, glucagon) were drawn at −10 min and at steady state. After at least 60 min of hyperinsulinemic-euglycemia, a CT scout and a low-dose CT scan were performed to localize the cardiac region and to correct subsequent emission data for tissue attenuation (75 mA, 120 kV, 0.938 pitch, 0.5 s of rotation time and 5 mm of slice thickness). Then, FDG (185 MBq) was infused over 15 s, and a 50-min dynamic scan was carried out (framing 12 × 10 s, 3 × 20 s, 4 × 30 s, 5 × 60 s, 8 × 150 s, 4 × 300 s). PET images were corrected for detector efficiency (normalization), system dead time, random coincidences, scatter, and attenuation, and reconstructed with an ordered subsets expectation maximization (OSEM) iterative algorithm, using 2 iterations and 21 subsets, applying point of spread function (PSF) correction and time of flight (TOF). A post-reconstruction Gaussian filtering with 3-mm full width at half maximum (FWHM) was applied. A 200 × 200 matrix size, zoom 2, was used for dynamic images. FDG image analysis was performed using the Cardiac PET Modeling Tool (PCARDP) software (version 4.2 by PMOD Technologies LLC). The full dynamic study was used for MGU calculation, regions of interest (ROIs) were drawn on the short axis images of the LV and the arterial input function was extracted from a volume of interest (VOI) placed in the LV cavity. Myocardial glucose fractional uptake rates were estimated using Patlak graphical analysis [20], usually applied for the analysis of FDG, which can be modeled as a 2-tissue compartment model with K4 = 0. The measured PET activity is divided by plasma activity and plotted at a “normalized time” (integral of input curve from injection divided by instantaneous plasma activity). A lumped constant of 1.0 and the plasma glucose level of the patient were entered and MGU (μmol/100 g/min) was obtained from the regression slope MGU = slope * plasma glucose/lumped constant.

### ***Resting and stress ***^***13***^***N-ammonia myocardial perfusion PET/CT***

Patients were studied according to the European Association of Nuclear Medicine (EANM) procedural guidelines for PET/CT quantitative myocardial perfusion imaging with ^13^ N-ammonia at rest and during pharmacologic stress (370 + 370 MBq) with adenosine (140 µg/kg/min for 6 min) [21]. PET images were reconstructed as described above. The dynamic list sequence was reframed for quantitative assessment of MBF measurements (framing: 10 × 5 s, 1 × 10 s, 6 × 30 s, 1 × 360 s) and 16-bin ECG-gated myocardial perfusion analysis for LVEF measurement. Stress and resting MBF were computed from the dynamic stress and rest imaging series, respectively, using a commercially available software (syngo.PET Myocardial Blood Flow by Siemens Healthcare). Quantitative perfusion estimates were derived as follows: short axis orientation procedure, LV contour definition, calculation of the time-activity curves and fitting of a 2-compartment kinetic model for ^13^N-ammonia to generate stress and resting MBF values in ml/g/min. To account for changes in resting MBF caused by cardiac workload, additional resting MBF values were obtained by correcting for rate-pressure product (RPP), an index of myocardial oxygen consumption, using the following formula: corrected resting MBF = (resting MBF/RPP) × 10^4^. Coronary flow reserve (CFR) was calculated as the ratio of stress to resting MBF. Corrected CFR values were also calculated using resting corrected MBF.

### Laboratory measurements

The following variables were measured at baseline and at the end of the study: Hematology: complete blood cell count, glycated hemoglobin; Serum chemistry: aspartate, aminotransferase (AST, SGOT), alanine aminotransferase (ALT, SGPT), alkaline phosphatase (ALK-P), creatine kinase (Creatine Phosphokinase, CK, CPK), serum creatinine (Scr), electrolytes – sodium, potassium, chloride, calcium, albumin, uric acid; Urinalysis: pH, protein, glucose, leukocyte esterase, blood, Microscopy if dipstick positive for blood or leukocyte esterase.

## Statistical analysis

Considering an expected delta between the two groups (dapa group vs placebo group, change from baseline) on MGU of 8.7 μmol/100 g/min and a standard deviation of 9.9, 23 patients per treatment group are considered a sufficient number to reject the null hypothesis that the population means of the two groups are equal with a power of 80% and an alpha of 0.05. Assuming a 10% loss in each group due to protocol violations/loss to follow-up, we estimate that the total number should be 26 patients per group. The details of sample size estimation and statistical analysis are available in the study design [18] and supplemental material. Data are presented as mean ± SEM or median (95% Confidence Interval, CI) as appropriate. Data were examined for normal distribution. Parametric and/or non-parametric tests were used, as appropriate. Within group differences were assessed using the t-test (or equivalent non-parametric test) for paired data; between group differences were assessed using the t test (or equivalent non-parametric test) for unpaired data. In addition, tests for repeated measurements were used to account for treatment versus group effects and interactions**.**

## Results

### Study population

A total of 16 patients were included in the study and randomized to receive dapagliflozin (n = 8) or placebo (n = 8) (Additional file 1: Fig. S2).

In one patient, all FDG data were excluded from the analysis because post-treatment FDG PET/CT images were not evaluable while pre- and post- myocardial perfusion data were considered. Patient compliance was generally high in both groups. No study drug-related severe adverse events were observed.

Demographic and baseline characteristics of each treatment group are summarized in Table 1.

**Table 1:** Baseline characteristics

**Baseline characteristics**	Placebo (n = 8)	Dapagliflozin 10 mg (n = 8)	P value
Male sex, N (%)	5 (62.5)	8 (100)	
Age, years	67 ± 2.2	66 ± 2.6	P = 0.7
Diabetes duration, years	8.25 ± 0.6	6.3 ± 1.0	P = 0.2
HbAlc, %	8.1 ± 0.2	7.8 ± 0.1	P = 0.45
Fasting glycemia (mg/dl)	136 ± 17	140 ± 12	P = 0.5
C-peptide (ng/ml)	1.4 ± 0.3	1.7 ± 0.2	P = 0.6
BMI, kg/m^2^	28.5 ± 1.0	27.3 ± 1.1	P = 0.3
Body weight, kg	81.8 ± 4.7	81.8 ± 2.6	P > 0.9
Heart rate, bpm	61 ± 4	65 ± 4	P = 0.9
Systolic BP, mmHg	135 ± 5.2	143 ± 5.1	P = 0.2
Diastolic BP, mmHg	71 ± 3.8	68 ± 4.4	P = 0.4
CAD (previous PCl/no PCI)	4/4	3/5	P = 0.3
eGFR (ml/min)	90.6 ± 3.1	85.8 ± 6.2	P = 0.5
*Medications*			
Metformin	8 (100)	7 (87.5)	
DPP- 4i	4 (50)	3 (37.5)	
GLP-1RA	2 (25)	1 (12.5)	
Basal insulin	2 (25)	3 (37.5)	
Sulfonylurea	2 (25)	1(12.5)	

Data are mean ± SEM. HbA1c, glycated hemoglobin; BMI, body mass index; CAD, coronary artery disease; eGFR, estimated glomerular filtration rate

The groups were generally well balanced.

### Primary and main secondary endpoints

**Fig. 1:**
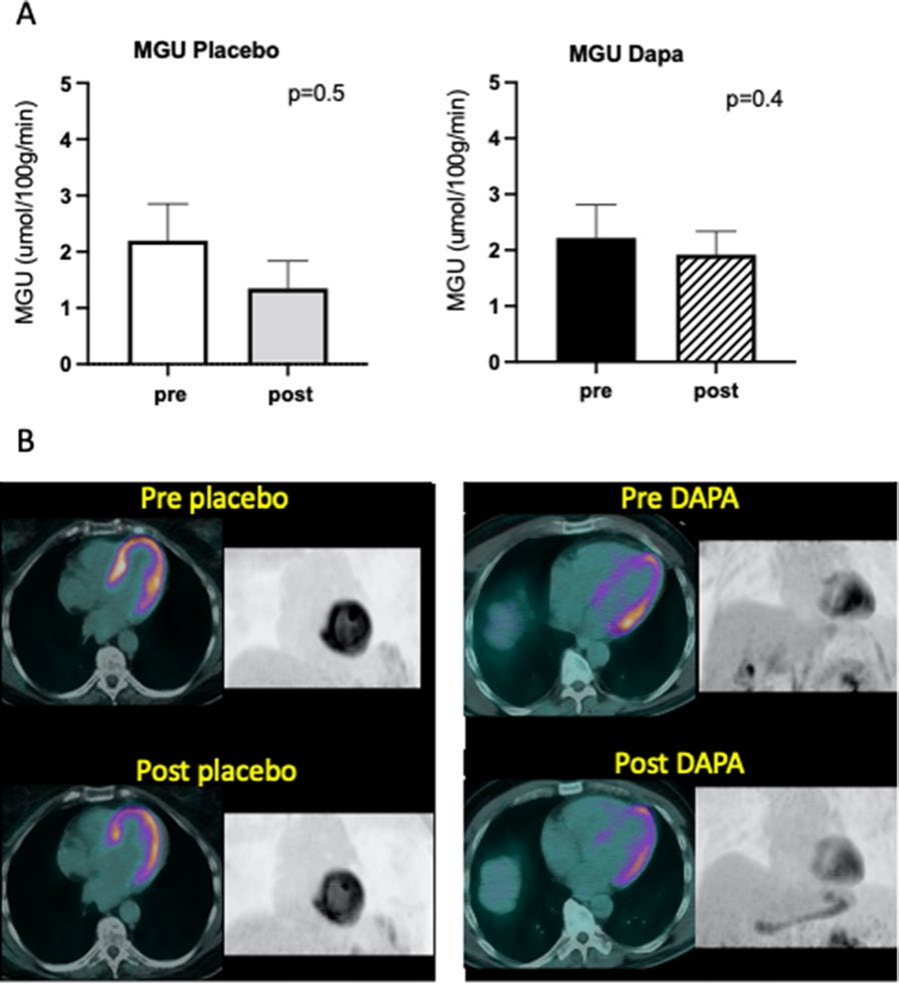
Myocardial glucose uptake (MGU). **A** Data are mean ± SEM; **B** FDG PET/CT images during euglycemic hyperinsulinemic clamp of two representative cases: fused and 3D maximum intensity projection anterior views pre- and post-placebo and pre- and post-dapagliflozin, respectively

There was no significant change in MGU during euglycemic hyperinsulinemic clamp in the dapagliflozin group (2.22 ± 0.59 vs 1.92 ± 0.42 μmol/100 g/min, p = 0.41) compared with the placebo group (2.00 ± 0.55 vs 1.60 ± 0.45, p = 0.5; Fig. 1A, B).

**Fig. 2:**
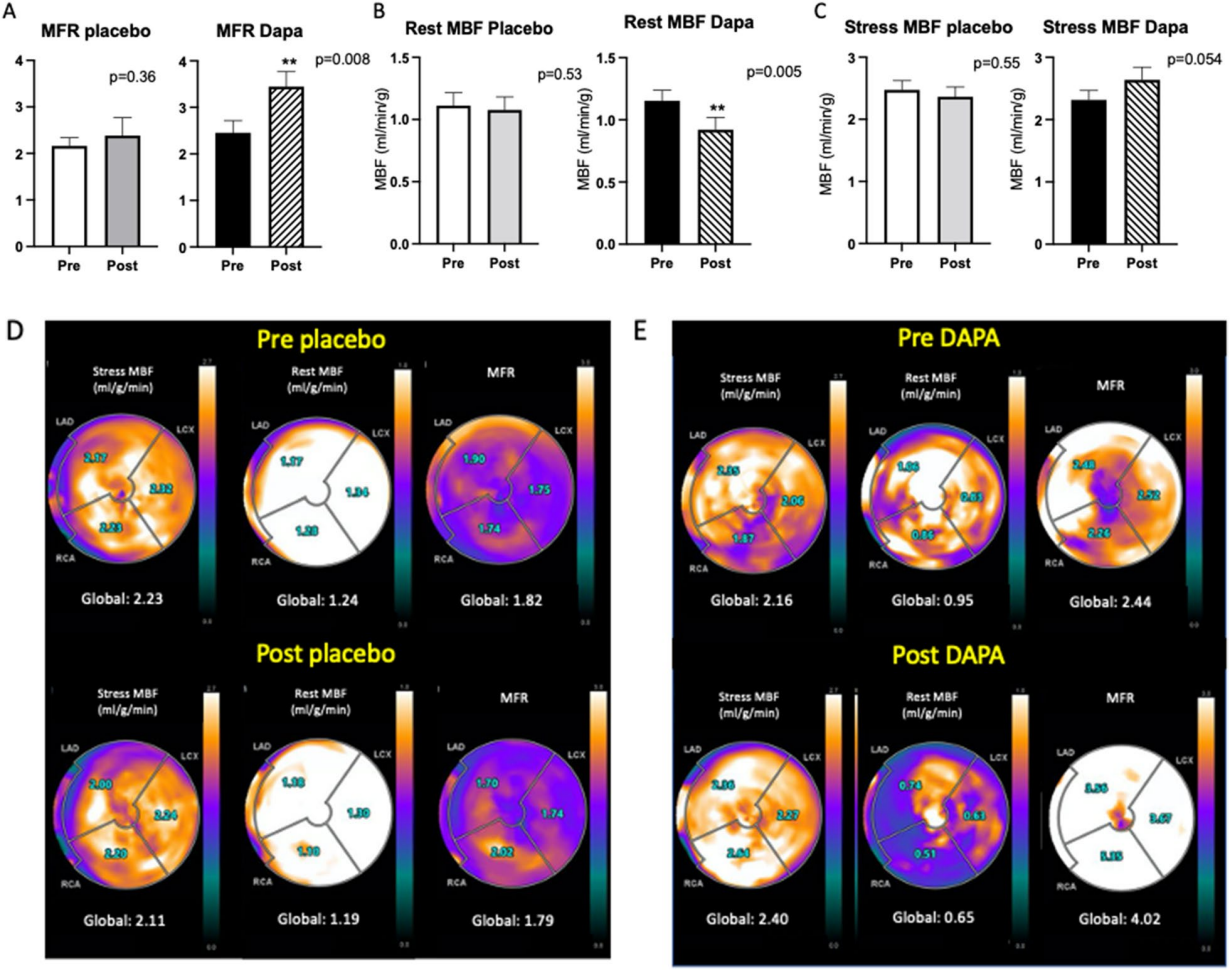
Myocardial perfusion: Myocardial flow reserve (**A**); resting myocardial blood flow (MBF) corrected for rate pressure product (**B**); stress myocardial blood flow (**C**); Data are mean ± SEM; **p < 0.01. Myocardial perfusion PET images of two representative cases: regional and global myocardial blood flow (MBF) and myocardial flow reserve (MFR) quantification at rest and during adenosine stress pre- and post-placebo (**D**) and pre- and post-dapagliflozin (**E**), respectively

Patients treated with dapagliflozin showed a significant improvement in MFR (2.56 ± 0.26 vs 3.59 ± 0.35; p = 0.006) compared with the placebo group (2.34 ± 0.21 vs 2.38 ± 0.24; p = 0.81; p for interaction = 0.001). Correction for resting RPP confirmed MFR increment in the dapagliflozin group (2.22 ± 0.25 vs 3.23 ± 0.4; p = 0.008; p for interaction = 0.019; Fig. 2A). Resting MBF was significantly lower in the dapagliflozin group, even after correction for resting RPP (1.15 ± 0.09 vs 0.92 ± 0.10 mL/min/g; p = 0.005) compared with the placebo group (1.20 ± 0.10 vs 1.18 ± 0.17 mL/min/g, p for interaction = 0.045; Fig. 2B). There was a trend toward an increase in stress MBF in the dapagliflozin group (2.32 ± 0.15 vs 2.64 ± 0.20 mL/min/g) compared with the placebo group (2.47 ± 0.15 vs 2.36 ± 0.16 mL/min/g; p = 0.054; Fig. 2C).

Both rest and stress LVEF did not significantly change in the DAPA group (60 ± 4% vs 63 ± 5% at rest and 61 ± 4% vs 64 ± 4% during pharmacological stress, p = 0.27 and p = 0.11 respectively) compared with the placebo (59 ± 3% vs 60 ± 3% at rest and 60 ± 3% vs 60 ± 4% during pharmacological stress, p = 0.77 and p = 0.66 respectively).

### Systemic metabolic effects of dapagliflozin and glycemic control

There was a numerical increase of WBGU during euglycemic hyperinsulinemic clamp in the dapagliflozin group (3.0 ± 0.53 vs 4.1 ± 0.52 mg/kg/min, p = 0.06; Additional file 1: Fig. S3). HbA1c significantly decreased after treatment in the dapagliflozin group (7.8 ± 0.2% vs 7.1 ± 0.2%, p = 0.0003). There were no significant changes in other clinical and laboratory parameters after dapagliflozin treatment.

## Discussion

This phase III, single-center, randomized, two-arm, parallel-group, double-blind, placebo-controlled study showed that, in T2D patients, 4 weeks of treatment with dapagliflozin increased myocardial flow reserve (MFR) and reduced resting myocardial blood flow (MBF), which was significant even after correction for cardiac workload. In addition, no effect on myocardial glucose uptake (MGU) was observed after treatment with dapagliflozin, while there was a numerical increase in whole body glucose uptake (WBGU) as measured directly with PET during the hyperinsulinemic euglycemic clamp.

Our results on resting MBF confirm those of previous studies using myocardial perfusion imaging with PET in T2D patients treated with SGLT-2i and provide new evidence on MFR. Lauritsen et al. [22] reported that 4 weeks of treatment with empagliflozin significantly reduced resting MBF, even after adjustment for cardiac workload, but did not significantly affect MFR. Oldgren et al. [23] also described a not significant decrease in resting myocardial perfusion after 6 weeks of dapagliflozin treatment but they did not measure MFR. In the study by Jürgens et al. [24], resting MBF decreased after 13 weeks of treatment with empagliflozin even in patients with T2D and high CV disease risk while MFR did not change significantly. Our study demonstrates, for the first time, a significant increase in MFR (stress/rest MBF) in a diabetic population after SGLT-2i treatment. This improvement was due to the combination of a significant reduction in resting MBF and an increase in stress MBF, which was of borderline statistical significance.

### The effect of SGLT-2i on myocardial glucose uptake

We had initially hypothesized [18] that treatment with SGLT-2i in patients with T2D affects cardiomyocyte metabolism via the glucose substrate, assessed by MGU measurement during hyperinsulinemic euglycemic clamp, and MBF. However, our results did not support the initial hypothesis on the effect on myocardial insulin sensitivity, as MGU did not change significantly after 4 weeks of dapagliflozin treatment. This is in line with the study published by Latva-Rasku and colleagues, which demonstrated that 8-week treatment with dapagliflozin does not affect tissue insulin sensitivity and MGU as measured with PET in T2D patients [25], but contrasts with other studies which instead described an increase in insulin sensitivity [26–28]. Succurro et al. [29] recently demonstrated that T2D patients randomized to empagliflozin showed a significant reduction in MGU during hyperinsulinemic euglycemic clamp after 26 weeks of treatment as compared to patients randomized to glimepiride who showed an increase in MGU. Empagliflozin treatment was also associated with a significant increase in WBGU compared to glimepiride, which induced a reduction in WBGU.

However, our finding of a significant improvement in MFR and resting MBF in T2D patients after SGLT-2i, even though not associated with MGU changes, does not exclude the metabolic hypothesis of a substrate shift in favor of ketones and/or an increase in free fatty acid (FFA) utilization that may be associated with an improvement in myocardial perfusion. Improvement in FFA oxidation has been largely demonstrated in whole body studies [27]. This hypothesis is confirmed by the significant decrease in fat mass induced by SGLT-2i [30]. Nevertheless, two previous studies demonstrated that SGLT-2i did not affect myocardial FFA uptake in T2D patients without HF as measured by PET [22, 31] while FFA uptake was significantly increased in the liver [31].

### The effect of SGLT-2i on myocardial flow reserve and the improvement in coronary microvascular dysfunction hypothesis

One of the major advantages of PET is that it can measure MFR, the ratio of MBF during near maximal coronary vasodilation to rest MBF, an integrated measure of flow through both the large epicardial coronary arteries and the microcirculation. In absence of obstructive coronary artery disease, as in our study population, the MFR may reflect the coronary microcirculation function. [32]. There are many alternative hypotheses that could explain the reduction in resting MBF after treatment with dapagliflozin, even after correction for rate-pressure product, such as a more efficient use of oxygen in myocytes, a reduction in myocardial oxygen consumption due to a reduction in contractility or an improvement in coronary microvascular dysfunction secondary to the chronic proinflammatory state of T2D [33, 34].

Previous studies have shown that myocardial function did not significantly change after dapagliflozin [31] and myocardial oxygen consumption was not significantly reduced by SGLT-2i [22, 31]. Therefore, the observed increase in MBF does not seem to be due to a reduction in myocardial oxygen consumption or contractility. An improvement in coronary microvascular dysfunction seems, therefore, a more appropriate interpretation of our data, and it might also help explain the nominal increase in stress MBF observed with dapagliflozin. Still, we can hypothesize that the positive effects of dapagliflozin treatment on MFR and resting MBF could be, at least in part, due to the action of dapagliflozin on the coronary endothelium, which results in a lower degree of endothelial inflammation/dysfunction and consequently less fibrosis, which, in turn, improves myocardial oxygenation/nutrient delivery. This hypothesis is supported by recently published in vitro and pre-clinical studies on SGLT-2i and endothelial cells showing the positive effect of SGLT-2i on the myocardium, possibly targeting the coronary endothelium [35–38]. Moreover, the reduction in myocardial glucose overflow (i.e., glucose toxicity) may lead to reduced myocardial inflammation, which is mainly caused by the decrease in myocardial glucose uptake (due to hyperglycemia per se), which could also support the beneficial effects of SGLT-2i on coronary microvascular dysfunction [39, 40]. Finally, possible mechanisms of action of SGLT-2i are continuously being investigated. Some of these are independent of the hypoglycemic effect of SGLT-2i and could be associated with the improvement in micro- and macrovascular endothelial function in T2D patients such as the increase in circulating provascular progenitor cells [41] and the increase in flow-mediated dilation due to the increase in nitric oxide production [42]. In addition, the vasodilatory action of SGLT-2i treatment by the inhibition of Na + /H + exchanger in cardiomyocytes [43] and by the selective stimulation of KV7 ion channels in arterial smooth muscle cells of resistance mesenteric arteries [44] has been recently reported in pre-clinical models.

In addition to the vasodilatory action of SGLT-2i treatment, accumulating evidence suggests that dapagliflozin reduces cardiac remodeling by regulating the TGF-β1/Smad signaling in a non-glucose-lowering dependent manner [45] and attenuates advanced glycation end product induced inflammation and apoptosis in diabetic nephropathy through AMPK-mTOR mediated autophagy pathway [46].

Our results, together with the above-mentioned data, have important potential clinical implications since endothelial dysfunction is a well-known potent marker of cardiovascular risk [34], and its attenuation may explain the cardioprotective effect of SGLT-2i beyond the hypoglycemic effect and possibly through mechanisms that are partly independent of glucose. However, the precise impact and mechanisms of SGLT-2i still need further research.

### Limitations and strengths of the study

The present study has some limitations. Because of its complex exploratory design and restricted inclusion criteria, it was relatively small: a substantial number of potential participants refused to enter the study due to its complexity and fear of being in hospital during the COVID-19 pandemic. A larger sample size might have revealed additional or even substantial differences, making the trends we observed statistically significant (i.e., stress MBF and WBGU increase in dapagliflozin group).

On the other hand, we studied the early effects of SGLT-2i on myocardial metabolism and perfusion using highly sophisticated and gold standard methods to assess insulin sensitivity (euglycemic hyperinsulinemic clamp), myocardial and whole body metabolism (glucose uptake calculated by FDG PET/CT during euglycemic hyperinsulinemic clamp), MBF and MFR (by PET/CT with 13N-ammonia) in a highly selected study population (T2D patients with a narrow HbA1c range and CAD not requiring revascularization or clinically stable after PCI).

## Conclusions

The present study provides evidence that dapagliflozin treatment does not affect myocardial glucose uptake in patients with T2D without obstructive coronary artery disease, whereas a significant increase in myocardial flow reserve was observed. We speculate that this increase might be caused by an improvement in coronary microvascular dysfunction, thus providing another potential explanation for the CV benefits obtained with treatment with SGLT-2i. In particular, SGLT-2i render patients more resistant to the detrimental effects of obstructive coronary atherosclerosis by increasing MFR, probably by improving coronary microvascular dysfunction. Further studies are warranted to investigate these complex and interconnected pathophysiological mechanisms.

## Supplementary Information

**Additional file 1. **SUPPLEMENTARY DATA

## Data Availability

The datasets generated and/or analyzed during the current study are available from the corresponding author on reasonable request.

## Abbreviations

CAD: Coronary artery disease
HF: Heart failure
LVEF: Left ventricular ejection fraction
MBF: Myocardial blood flow
MFR: Myocardial flow reserve
MGU: Myocardial glucose uptake
PET: Positron emission tomography
RAS: Renin-angiotensin system
SGLT-2i: Sodium-glucose cotransporter-2 inhibitors
T2D: Type 2 diabetes
WBGU: Whole body glucose uptake
