# Supplementary material for: Dapagliflozin improves myocardial flow reserve in patients with type 2 diabetes: the DAPAHEART Trial: a preliminary report
Source: Cardiovasc Diabetol. 2022 Sep 3;21:173. doi: 10.1186/s12933-022-01607-4 (PMC9440459; doi:10.1186/s12933-022-01607-4)
Supplement: Supplementary file 1 — Additional file 1. SUPPLEMENTARY DATA [file 12933_2022_1607_MOESM1_ESM.docx]

**
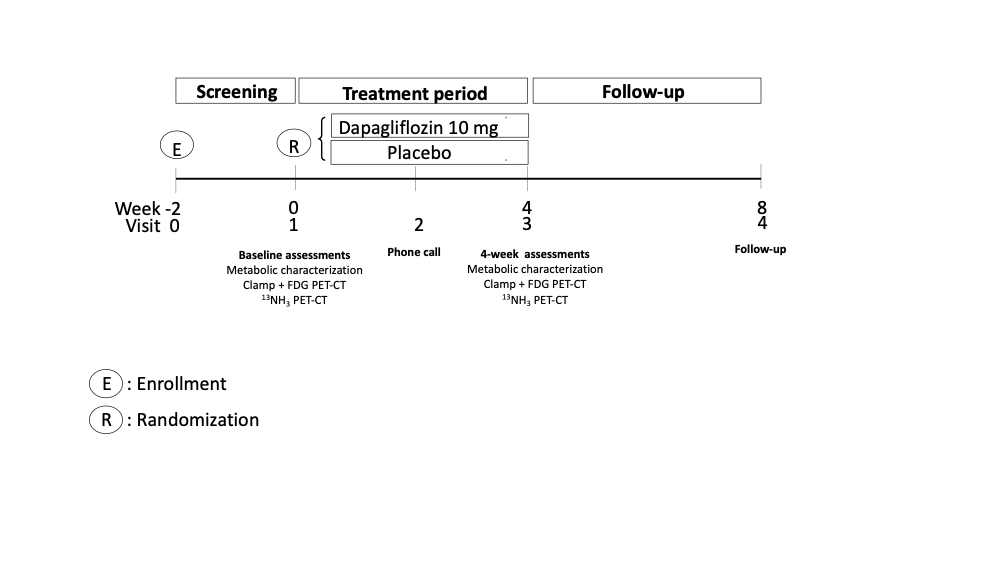
 Fig. S1:** Trial design. *Original with permission from G.P. Sorice et al. 2021, DOI: 10.1007/s13300-021-01083-1*

**
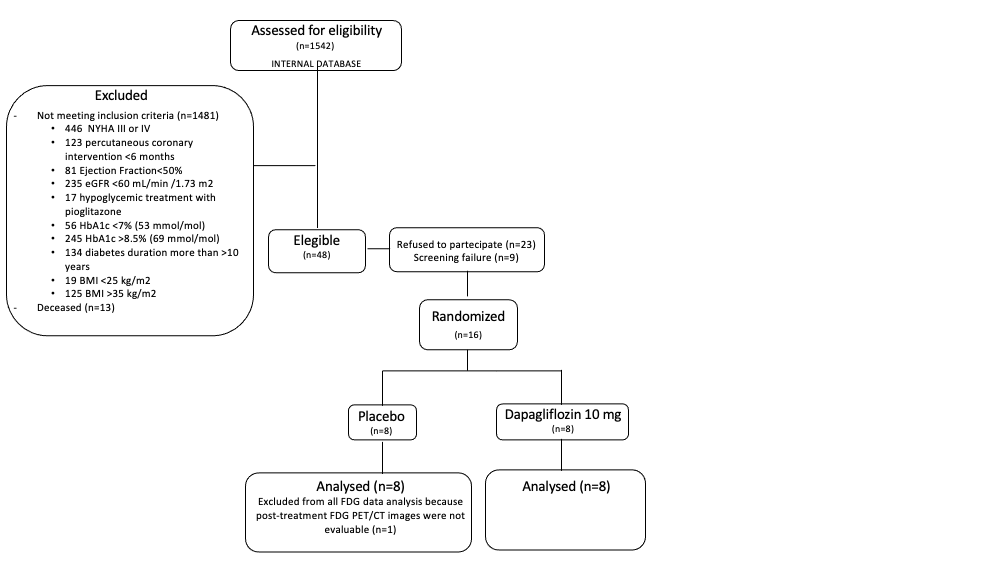
 Fig. S2:** Flowchart of study participants.


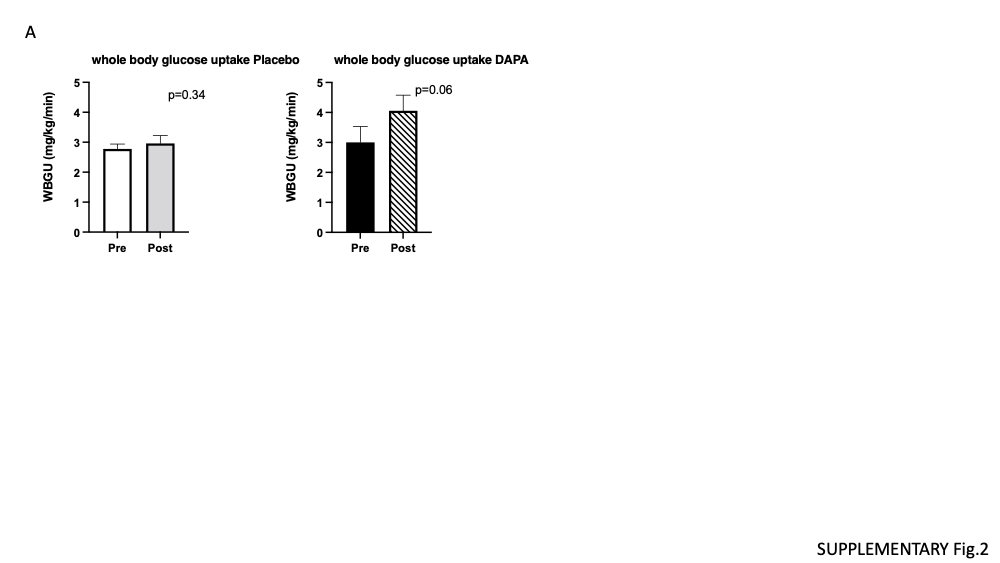


**Fig.S3:** Whole body glucose uptake: Data are mean ± SEM; *p<0.05
